# Nomogram Model for Prediction of SARS-CoV-2 Breakthrough Infection in Fujian: A Case–Control Real-World Study

**DOI:** 10.3389/fcimb.2022.932204

**Published:** 2022-06-23

**Authors:** Tianbin Chen, Yongbin Zeng, Di Yang, Wenjing Ye, Jiawei Zhang, Caorui Lin, Yihao Huang, Yucheng Ye, Jianwen Li, Qishui Ou, Jinming Li, Can Liu

**Affiliations:** ^1^ Department of Laboratory Medicine, Gene Diagnosis Research Center, The First Affiliated Hospital of Fujian Medical University, Fuzhou, China; ^2^ Fujian Key Laboratory of Laboratory Medicine, The First Affiliated Hospital of Fujian Medical University, Fuzhou, China; ^3^ Software Engineering Institution, East China Normal University, Shanghai, China; ^4^ Department of Emergency Response and Epidemic Situation Monitoring, Fujian Provincial Center for Disease Control and Prevention, Fuzhou, China; ^5^ National Center for Clinical Laboratories, Institute of Geriatric Medicine, Chinese Academy of Medical Sciences, Beijing Hospital/National Center of Gerontology, Beijing, China

**Keywords:** SARS-CoV-2 breakthrough infection, vaccinated individuals, nomogram, prediction, model

## Abstract

SARS-CoV-2 breakthrough infections have been reported because of the reduced efficacy of vaccines against the emerging variants globally. However, an accurate model to predict SARS-CoV-2 breakthrough infection is still lacking. In this retrospective study, 6,189 vaccinated individuals, consisting of SARS-CoV-2 test-positive cases (n = 219) and test-negative controls (n = 5970) during the outbreak of the Delta variant in September 2021 in Xiamen and Putian cities, Fujian province of China, were included. The vaccinated individuals were randomly split into a training (70%) cohort and a validation (30%) cohort. In the training cohort, a visualized nomogram was built based on the stepwise multivariate logistic regression. The area under the curve (AUC) of the nomogram in the training and validation cohorts was 0.819 (95% CI, 0.780–0.858) and 0.838 (95% CI, 0.778–0.897). The calibration curves for the probability of SARS-CoV-2 breakthrough infection showed optimal agreement between prediction by nomogram and actual observation. Decision curves indicated that nomogram conferred high clinical net benefit. In conclusion, a nomogram model for predicting SARS-CoV-2 breakthrough infection based on the real-world setting was successfully constructed, which will be helpful in the management of SARS-CoV-2 breakthrough infection.

## Introduction

Two inactivated vaccines (Sinovac and Sinopharm), widely used in mainland China, are highly effective in preventing COVID-19, hospitalization, intensive care unit admission, and COVID-19-related deaths (Jara et al., 2021). However, as SARS-CoV-2 variants are emerging globally, the efficacy of the vaccines might reduce (Brosh-Nissimov et al., 2021). In a real-world study in Guangzhou City, China, in May 2021, the efficacy of two-dose vaccination was 59.0% against the Delta variant (Li et al., 2021). More and more SARS-CoV-2 breakthrough infection has been reported around the world (Rana et al., 2021; Brown et al., 2021; He et al., 2021; Karim and Karim, 2021), highlighting the need to identify prominent risk factors correlated with SARS-CoV-2 breakthrough infection in vaccinated individuals.

Evidence from randomized controlled trials of mRNA-1273 (Gilbert et al., 2021) and ChAdOx1 (Feng et al., 2021) vaccination indicated a higher risk for SARS-CoV-2 breakthrough infection among persons with lower neutralizing, spike, or receptor-binding domain (RBD) titers in the early breakthrough infection period. Real-world data suggested that among fully vaccinated healthcare workers, the occurrence of SARS-CoV-2 breakthrough infection was highly correlated with neutralizing antibody titers during the peri-infection period (Bergwerk et al., 2021), and in vaccinated patients receiving dialysis low levels of circulating RBD antibody, it was associated with higher risk for breakthrough infection (Anand et al., 2021). Meanwhile, partially vaccinated individuals without obesity (body mass index (BMI) < 30 kg/m^2^) had lower odds of SARS-CoV-2 breakthrough infection (Antonelli et al., 2021), while a history of contact with a confirmed positive case and presence of symptoms were major risk factors for SARS-CoV-2 breakthrough infection in fully vaccinated individuals (Alishaq et al., 2021). It can be concluded from previous studies that risk factors of SARS-CoV-2 breakthrough infection may vary due to the different vaccine brands, vaccinated types, demographics, or underlying conditions. Therefore, it is necessary to conduct more studies to identify SARS-CoV-2 breakthrough infection risk factors, and it is meaningful to construct models based on those risk factors to predict SARS-CoV-2 breakthrough infection.

During the recent and first SARS-CoV-2 Delta variant outbreak in Fujian province, mainland China, 10,961 close contacts including 471 (4.30%) COVID-19 cases have been identified as linked to the first patient possibly transmitted by an imported case. In total, 8,345 (76.13%) of them, 891 (8.12%) close contacts who received the first-dose vaccination (partially vaccinated) and 7,454 (67.08%) close contacts who received the second-dose vaccination (fully vaccinated), have been vaccinated, and most of them are vaccinated by Sinovac and Sinopharm. Further, there are 6,189 close contacts (partially or fully vaccinated) whose index cases’ vaccinated information and SARS-CoV-2 RT-PCR testing results are complete. This real-world setting might provide a good opportunity to find risk factors and establish a reliable model to predict SARS-CoV-2 breakthrough infection against the Delta variant, which will help us distinguish vaccinated individuals who are at high risk of breakthrough infection and develop guidance to augment their protection, either by continued social distancing or by additional active or passive vaccinations.

## Methods

### Study Design and Population

The demographic (age, sex, occupation, etc.), SARS-CoV-2 vaccination (date of vaccination and manufacturer), epidemiology (exposure date, relationship with other cases and contacts), and clinical characteristics (symptoms, severity classification, date of onset, and SARS-CoV-2 RT-PCR assay results) of 10,963 close contacts and index cases were collected by experts in Fujian Provincial Center for Disease Control and Prevention (FJCDC). A confirmed case with SARS-CoV-2 breakthrough infection was defined as a positive result of repeat RT-PCR assays for nasal and pharyngeal swab specimens.

In this study, the vaccination time of vaccinated individuals with SARS-CoV-2 breakthrough infection, defined as the first dose time or the second dose time, was calculated from the vaccination date to the date of RT-PCR testing positive result, while the vaccination time of non-SARS-CoV-2 breakthrough infection vaccinated individuals were calculated from vaccination date to September 15, 2021, when the epidemic outbreak of SARS-CoV-2 Delta variant reached to peak in Fujian. If cases were not vaccinated, we defined the vaccinated brand as unvaccinated and the vaccinated time as zero.

Because 2 weeks was required to form protective effects against SARS-CoV-2 infections, we defined the first-dose vaccination (partially vaccinated) and second-dose vaccination (fully vaccinated) as vaccinated time having elapsed for more than 14 days. Otherwise, study participants would be deemed unvaccinated despite that they had received the first dose of vaccination or deemed to have received the first dose of vaccination only although they had received the second dose.

**Figure 1:**
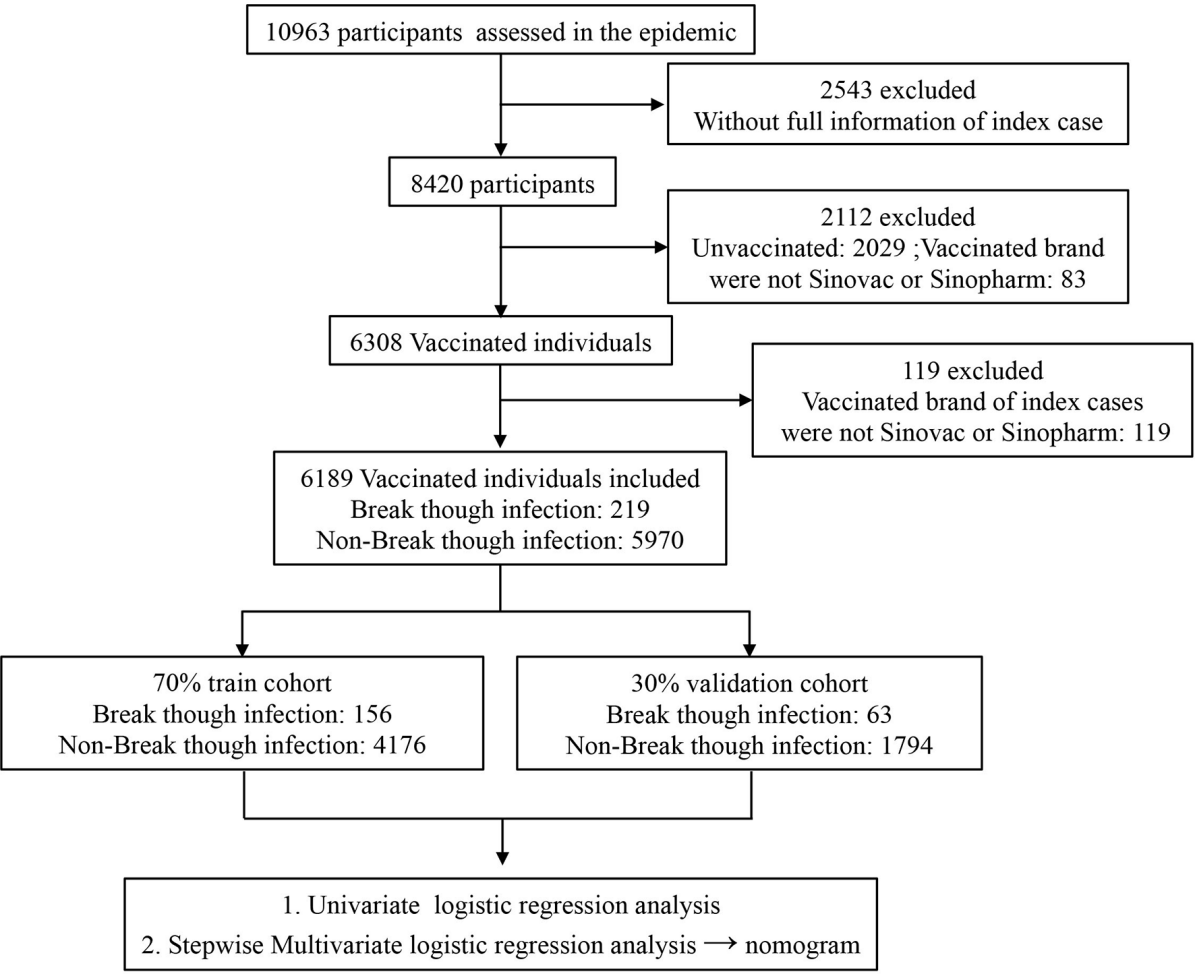
Flowchart of selection of study participants.

In total, 6,189 vaccinated individuals whose index cases’ vaccinated information and SARS-CoV-2 RT-PCR testing results were complete were recruited including 219 (3.54%) with SARS-CoV-2 breakthrough infection and 5,970 (96.46%) without breakthrough infection. The recruited vaccinated individuals were randomly grouped into a training (70%) cohort and a validation (30%) cohort to construct and evaluate the logistic regression model (**Figure 1**).

The study was approved by the Joint Prevention and Control Mechanism of the State Council.

### A Nomogram Construction

In the training cohort, univariate and multivariate logistic regression models *via* stepwise regression analysis were constructed. We further simplified the complex logistic regression model into a visualized nomogram by using the *rms* package of R. Subsequently, the efficiency of the visualized nomogram was evaluated by receiver operating characteristic (ROC) curve, calibration curve, and decision curve in both training and validation cohorts. The ROC was used to assess the discriminative ability of the nomogram and then the area under the curve (AUC). ROC analysis was used to calculate the optimal cutoff values that were determined by maximizing Youden’s index. The accuracy of the optimal cutoff value was assessed by sensitivity and specificity. A calibration curve was used to compare the association between actual outcomes and predicted probabilities. Decision curve analysis (DCA) was performed by calculating the net benefits for a range of threshold probabilities to evaluate the clinical utility of the nomogram (Vickers and Elkin, 2006; Pan et al., 2020).

### Statistical Analysis

Baseline continuous variables were expressed as means (SDs), and categorical variables were presented as frequency (%). Differences between the SARS-CoV-2 breakthrough infection group and the non-SARS-CoV-2 breakthrough infection group were used for the Wilcoxon test for continuous variables and the chi-squared test for categorical variables. Variable correlations were measured using Spearman’s correlation coefficient.

All *p*-values were two-sided, and *p* < 0.05 was regarded as significant. All statistical analyses, modeling, and plotting were performed with R (version 4.1.0 http://www.r-project.org).

## Result

### Patient Characteristics

The demographic characteristics of 6,189 partially or fully vaccinated individuals with or without SARS-CoV-2 breakthrough infection are shown in **Table 1**. The 219 vaccinated individuals with SARS-CoV-2 breakthrough infection were much older than non-SARS-CoV-2 breakthrough infection vaccinated individuals (41.52 ± 13.03 vs 36.43 ± 14.14, *p* < 0.001). There were more women (59.4% vs 50.9%, *p* = 0.017) in the SARS-CoV-2 breakthrough infection group. Meanwhile, there was a significant difference in the first dose brand, the first dose time, the second dose brand, and the second dose time between vaccinated individuals with or without SARS-CoV-2 breakthrough infection as well as index cases implying the efficacy of different vaccination methods might vary.

**Table 1:** Characteristics of vaccinated individuals with or without SARS-CoV-2 breakthrough infection.

	Non-breakthrough infection	Breakthrough infection	*p*-Values
	n = 5,970	n = 219	
Age (years, %)			<0.001
<20	839 (14.1)	15 (6.8)	
20–30	1,124 (18.8)	18 (8.2)	
30–40	1,743 (29.2)	71 (32.4)	
40–50	1,204 (20.2)	68 (31.1)	
50–60	770 (12.9)	33 (15.1)	
>60	290 (4.9)	14 (6.4)	
Sex (%)			0.017
Female	3,039 (50.9)	130 (59.4)	
Male	2,931 (49.1)	89 (40.6)	
Vaccine situation (%)			0.458
Partially vaccinated	705 (11.8)	30 (13.7)	
Fully vaccinated	5,265 (88.2)	189 (86.3)	
First dose brand (%)			0.014
Sinovac	3,082 (51.6)	132 (60.3)	
Sinopharm	2,888 (48.4)	87 (39.7)	
Second dose brand (%)			0.009
Sinopharm	2,494 (41.8)	71 (32.4)	
Sinovac	3,137 (52.5)	129 (58.9)	
Unvaccinated	339 (5.7)	19 (8.7)	
First dose time (days, %)			<0.001
<60	1,854 (31.1)	62 (28.3)	
60–120	2,586 (43.3)	122 (55.7)	
>120	1,530 (25.6)	35 (16.0)	
Second dose time (days, %)			<0.001
<60	3,105 (52.0)	97 (44.3)	
60–120	1,883 (31.5)	109 (49.8)	
>120	982 (16.4)	13 (5.9)	
IC age (years, %)			<0.001
<20	1,057 (17.7)	36 (16.4)	
20–30	347 (5.8)	6 (2.7)	
30–40	1,686 (28.2)	123 (56.2)	
40–50	2,301 (38.5)	36 (16.4)	
50–60	398 (6.7)	8 (3.7)	
>60	181 (3.0)	10 (4.6)	
IC sex (%)			0.769
Female	3,318 (55.6)	119 (54.3)	
Male	2,652 (44.4)	100 (45.7)	0.769
IC vaccine situation (%)			0.767
Partially vaccinated	365 (6.1)	16 (7.3)	
Fully vaccinated	4,325 (72.4)	156 (71.2)	
Unvaccinated	1,280 (21.4)	47 (21.5)	
IC first dose brand (%)			<0.001
Sinopharm	2,143 (35.9)	128 (58.4)	
Sinovac	2,956 (49.5)	47 (21.5)	
Unvaccinated	871 (14.6)	44 (20.1)	
IC second dose brand (%)			<0.001
Sinopharm	2,042 (34.2)	26 (11.9)	
Sinovac	2,438 (40.8)	139 (63.5)	
Unvaccinated	1,490 (25.0)	54 (24.7)	
IC first dose time (days, %)			<0.001
<60	2,090 (35.0)	71 (32.4)	
60–120	2,063 (34.6)	132 (60.3)	
>120	1,817 (30.4)	16 (7.3)	
IC second dose time (days, %)			<0.001
<60	2,850 (47.7)	133 (60.7)	
60–120	1,980 (33.2)	82 (37.4)	
>120	1,140 (19.1)	4 (1.8)	
IC ORF1ab gene (Ct values)	24.24 (6.42)	21.54 (6.02)	<0.001
IC N gene (Ct values)	23.19 (6.56)	21.78 (5.59)	0.002

IC, index cases of vaccinated individuals.

To construct and evaluate the logistic regression model, those vaccinated individuals recruited were randomly split into a training (70%) cohort and a validation (30%) cohort. As shown in **Supplementary Table 1**, the difference of each variable between the training and validation cohorts was not significant, implying that the segmentation process was random and balanced. As shown in **Supplementary Figure 1**, the correlation between ORF1ab gene Ct value and N gene Ct values was significantly high (*R* = 0.958, *p* < 0.001) in index cases; to avoid collinearity and overfitting in a logistic regression model, only ORF1ab gene Ct values were included in the further analysis process in this study.

### Development and Validation of a Nomogram

**Figure 2:**
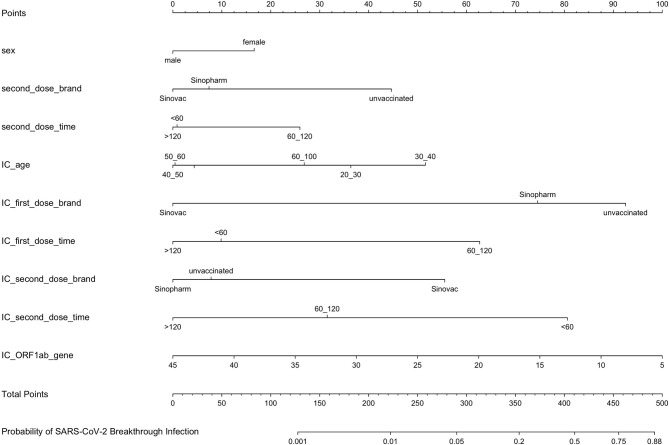
A predictive nomogram model for SARS-CoV-2 breakthrough infection established by independently associated risk factors from multivariate logistic regression. IC, index case of vaccinated individuals.

**Figure 3:**
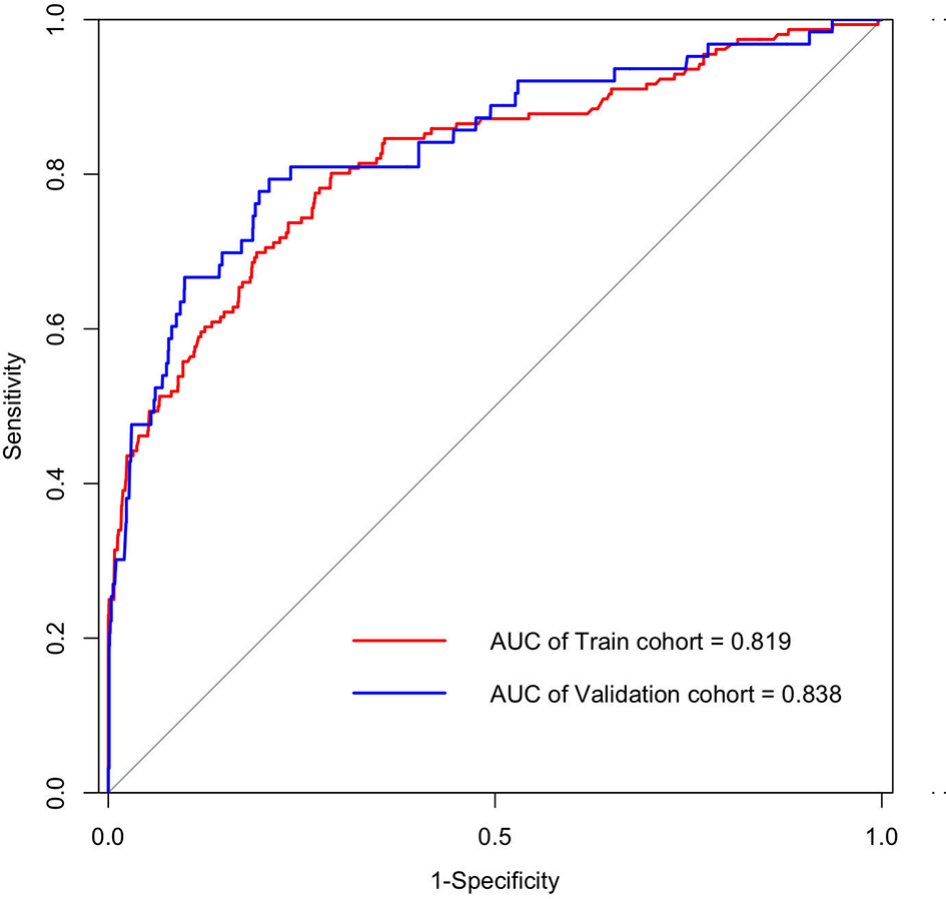
ROC curves of the nomogram for predicting SARS-CoV-2 breakthrough infection among vaccinated individuals in training cohort (AUC, 0.819; 95% CI, 0.780–0.858; sensitivity, 0.712; and specificity, 0.801 at the optimal cutoff of 0.027) and validation cohort (AUC, 0.838; 95% CI, 0.778–0.897; sensitivity, 0.792; and specificity, 0.794 at the optimal cutoff of 0.041). AUC, the area under the receiver operating characteristic (ROC) curve.

Univariate and multivariate logistic regression analysis results, presented in **Table 2**, show the reported odds ratio (95% CI), sex of vaccinated individuals sex (for female vs male, 1.500 [1.042–2.161], *p* = 0.029), vaccinated individuals’ second dose brand (for vs Sinopharm vs unvaccinated, 0.403 [0.193–0.839], *p* = 0.015; for vs Sinovac vs unvaccinated, 0.336 [0.193–0.839], *p* = 0.003), vaccinated individuals’ second dose time (for 60–120 vs <60 days, 1.846 [1.235–2.758], *p* = 0.003), age of index case (for 30–40 vs <20 years old, 3.170 [1.653–6.078], *p* = 0.001), first dose brand of index case (for Sinovac vs unvaccinated, 0.105 [0.042–0.259], *p* < 0.001), first dose time of index case (for 60–120 vs <60 days, 3.634 [1.809–7.301], *p* < 0.001), second dose brand of index case (for Sinovac vs unvaccinated, 3.208 [1.228–8.383], *p* = 0.017), second dose time of index case (for 60–120 vs <60 days, 0.302 [0.177–0.516], *p* < 0.001; for >120 vs < 60 days, 0.140 [0.030–0.649], *p* = 0.012), and ORF1ab gene Ct values of index case (0.941 [0.911–0.972], *p* < 0.001). The independently associated risk factors (sex, second dose brand and second dose time of vaccinated individuals and age, first dose brand, first dose time, second dose brand, second dose time, and ORF1ab gene Ct values of index case) in multivariate logistic regression were incorporated to establish a predictive nomogram model for SARS-CoV-2 breakthrough infection (**Figure 2**). Performance of this nomogram was assessed by ROC, calibration curve, and decision curve in both training cohort and validation cohort. The nomogram demonstrated good accuracy in estimating the risk of SARS-CoV-2 breakthrough infection, with AUCs in training and validation cohorts being 0.819 (95% CI, 0.780–0.858; sensitivity, 0.801; specificity, 0.712; positive predictive value, 0.094; negative predictive value, 0.989; accuracy, 0.715) and 0.839 (95% CI, 0.728–0.897; sensitivity, 0.794; specificity, 0.792; positive predictive value, 0.118; negative predictive value, 0.990; accuracy, 0.792) (**Table 3** and **Figure 3**).

**Table 2:** Univariate and multivariate logistic regression analyses of SARS-CoV-2 breakthrough infection in vaccinated individuals.

Variable	Univariate analysis		Multivariate analysis	
	OR (95% CI)	*p*-Value	OR (95% CI)	*p*-Value
Sex				
Male	Reference			
Female	1.709 (1.227–2.38)	0.002	1.500 (1.042–2.161)	0.029
Age (years)				
<20	Reference			
20–30	0.88 (0.378–2.05)	0.767		
30–40	2.499 (1.261–4.952)	0.009		
40–50	3.296 (1.654–6.567)	0.001		
50–60	2.674 (1.273–5.619)	0.009		
60–100	2.747 (1.1–6.86)	0.03		
First dose brand				
Sinopharm	Reference			
Sinovac	1.462 (1.054–2.028)	0.023		
First dose time (days)				
<60	Reference			
60–120	1.883 (1.279–2.773)	0.001		
>120	0.844 (0.505–1.412)	0.519		
Second dose brand				
Unvaccinated	Reference			
Sinopharm	0.533 (0.286–0.994)	0.048	0.403 (0.193–0.839)	0.015
Sinovac	0.722 (0.397–1.311)	0.284	0.336 (0.163–0.693)	0.003
Second dose time (days)				
<60	Reference			
60–120	2.127 (1.523–2.97)	<0.001	1.846 (1.235–2.758)	0.003
>120	0.489 (0.25–0.958)	0.037	0.979 (0.455–2.107)	0.957
IC sex				
Male	Reference			
Female	1.006 (0.729–1.388)	0.972		
IC age (years)				
<20	Reference			
20–30	0.749 (0.303–1.854)	0.532	2.184 (0.764–6.245)	0.145
30–40	2.363 (1.492–3.741)	<0.001	3.170 (1.653–6.078)	0.001
40–50	0.494 (0.282–0.867)	0.014	0.899 (0.427–1.892)	0.779
50–60	0.413 (0.142–1.201)	0.105	0.909 (0.285–2.897)	0.872
60–100	1.445 (0.579–3.605)	0.43	1.732 (0.616–4.871)	0.298
IC first dose brand				
Unvaccinated	Reference			
Sinopharm	1.414 (0.913–2.19)	0.12	0.645 (0.24–1.736)	0.385
Sinovac	0.356 (0.212–0.597)	<0.001	0.105 (0.042–0.259)	<0.001
IC first dose time (days)				
<60	Reference			
60–120	1.893 (1.333–2.688)	<0.001	3.634 (1.809–7.301)	<0.001
>120	0.272 (0.144–0.513)	<0.001	0.786 (0.275–2.245)	0.653
IC second dose brand				
Unvaccinated	Reference			
Sinopharm	0.398 (0.227–0.7)	0.001	0.826 (0.292–2.341)	0.720
Sinovac	1.763 (1.192–2.608)	0.005	3.208 (1.228–8.383)	0.017
IC second dose time (days)				
<60	Reference			
60–120	0.843 (0.603–1.179)	0.319	0.302 (0.177–0.516)	<0.001
>120	0.078 (0.025–0.248)	<0.001	0.140 (0.030–0.649)	0.012
IC ORF1ab gene (Ct values)	0.931 (0.907–0.956)	<0.001	0.941 (0.911–0.972)	<0.001

IC, index case of vaccinated individuals; Reference, the reference group in the univariate or multivariable logistic regression models.

**Table 3:** The performance of the nomogram for predicting SARS-CoV-2 breakthrough infection in vaccinated individuals.

Index	Training cohort	Validation cohort
	(n = 4,332)	(n = 1,857)
AUC (95% CI)	0.819 (0.780–0.858)	0.838 (0.778–0.897)
Sensitivity	0.801	0.794
Specificity	0.712	0.792
Positive predictive value	0.094	0.118
Negative predictive value	0.989	0.990
Recall	0.801	0.794
Accuracy	0.715	0.792

AUC, area under the curve.

**Figure 4:**
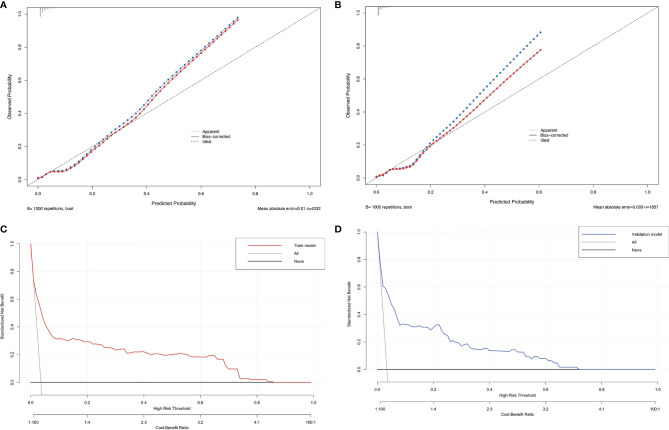
The calibration plot of the nomogram in the training **(A)** and validation cohorts **(B)**. Actual rate of SARS-CoV-2 breakthrough infection is shown on the y-axis, and the nomogram-predicted probability of SARS-CoV-2 breakthrough infection is shown on the x-axis. Decision curve compared the net clinical benefits of three scenarios in predicting the SARS-CoV-2 breakthrough infection probability: a perfect prediction model (gray line), no screen (horizontal solid black line), and screen based on the nomogram (red or blue line) in training cohort **(C)** and validation cohort **(D)**.

To evaluate the clinical applicability of our prediction nomogram, calibration curve and DCA were performed. In **Figures 4A, B**, the calibration plot for SARS-CoV-2 breakthrough infection probability showed an excellent agreement between the prediction by nomogram and actual observation in the training cohort and validation cohort, respectively, which indicated good calibration of the model.

DCA is a novel method for evaluating diagnostic and prognostic prediction models, which has some advantages over AUC (Vickers and Elkin, 2006). The DCA curves of nomogram showed a superior overall net benefit within the wide and practical ranges of threshold probabilities, which indicated that it had better clinical utility in predicting SARS-CoV-2 breakthrough infection in vaccinated individuals (**Figures 4C, D**).

## Discussion

The ongoing global COVID-19 pandemic has infected hundreds of millions of people over the world, and SARS-CoV-2 vaccines are currently the best defense against COVID-19 while being relatively safe in trial studies (Baden et al., 2021; Liu et al., 2021). However, SARS-CoV-2 breakthrough infections have been reported as the SARS-CoV-2 variants emerging globally (Brosh-Nissimov et al., 2021), especially with the emergence of the Omicron variant (Karim and Karim, 2021). Risk factors associated with COVID-19 breakthrough infections among vaccinated individuals have been identified in different scenarios (Bergwerk et al., 2021; Anand et al., 2021; Antonelli et al., 2021; Alishaq et al., 2021; Hacisuleyman et al., 2021), which would help us distinguish vaccinated individuals who are at high risk of SARS-CoV-2 breakthrough infection.

In this real-world cohort study, we conducted a retrospective analysis to identify risk factors associated with SARS-CoV-2 breakthrough infection and construct a nomogram model based on multivariate logistic regression analysis to predict SARS-CoV-2 breakthrough infection in vaccinated individuals to fight against the Delta variant.

In the nomogram model, if vaccinated individuals were female, partially vaccinated, or fully vaccinated with the second vaccination dose time between 60 and 120 days, the vaccinated individuals were more likely to experience breakthrough infection. In addition, when vaccinated individuals come into close contact with index cases who were unvaccinated (first dose brand: unvaccinated), partially vaccinated (first dose time was 60–120 days), or fully vaccinated while the second dose brand was Sinovac and the second dose time was <60 days, the probability of breakthrough infection was high. The ORF1ab Ct values of the index case contributed greatly to the nomogram; the lesser the ORF1ab Ct values, the higher the probability of breakthrough infection. Interestingly, when the age of the index case was 30–40 years, the odds ratio (3.170, *p* = 0.001) was much higher, which might be because index cases mainly came from local shoe and clothes factories where most workers were young. Consequently, it can be deduced that it is necessary to boost vaccination in vaccinated individuals against COVID-19, and unvaccinated index cases with lower ORF1ab Ct values would lead to more secondary attacks.

The nomogram had excellent performance metrics in both the training cohort and validation cohort: AUC (0.819 vs 0.838), sensitivity (0.801 vs 0.794), specificity (0.712 vs 0.792), negative predictive value (0.989 vs 0.990), accuracy (0.715 vs 0.792), and recall score (0.801 vs 0.794) were reasonably high. However, its positive predictive value (0.094 vs 0.118) was poor, which may be due to the low rate (3.54%, 219/6,189) of breakthrough infection in this cohort. Even though the positive predictive value was poor, considerably high AUC, negative predictive value, and recall score were achieved, implying that the nomogram would perform accurately in identifying vaccinated individuals with a high probability of breakthrough infection.

Compared with other risk factors like neutralizing antibody titers in the peri-infection period (Bergwerk et al., 2021) to predict SARS-CoV-2 breakthrough infection, risk factors in our nomogram model were conveniently retrieved at any time, making our model more applicable in real-world settings. Moreover, this nomogram model had a good performance and was easy to understand and interpret. Generally, our nomogram performed well in terms of discrimination, calibration, and clinical utility in predicting who was at high risk of SARS-CoV-2 breakthrough infection. To our knowledge, this is the first report of a quantitative nomogram model for predicting SARS-CoV-2 breakthrough infection. These findings represent invaluable data on vaccinated individuals obtained during the first COVID-19 Delta variant pandemic in Fujian and have important implications for the clinical management of such cases. In addition, as the Omicron variants are more transmissible than other variants and the virology and biology of Omicron variants are still limited (Wang and Powell, 2021; Chen et al., 2022), our nomogram is based on inactivated vaccines against Delta variants in a real-world setting and may provide an alternative strategy for predicting Omicron breakthrough infection.

In conclusion, we successfully constructed a nomogram model for predicting SARS-CoV-2 breakthrough infection based on the real-world setting, which will be helpful in the management of SARS-CoV-2 breakthrough infection.

## Limitations

Our study has several limitations. First, even though a cohort of 6,189 vaccinated individuals was recruited, the number of breakthrough infection cases (219) was relatively small. Second, the majority of the cohort was young and composed of vaccinated individuals with breakthrough infections who were mainly asymptomatic or mild. Thus, symptoms in some vaccinated individuals and index cases like fever, cough, sputum, nose stuffiness, or diarrhea, which may indicate COVID-19 or accelerate the spread of SARS-CoV-2, were not regarded as risk factors in this study. Third, some reported risk factors like neutralizing antibody titers in the peri-infection period or BMI were excluded as well because data were missing in most participants. Therefore, more risk factors and more participants should be included to improve the efficiency of the nomogram model, and multicenter data are needed to validate the accuracy of models in the future.

## Data Availability Statement

The raw data supporting the conclusions of this article will be available on reasonable request, without undue reservation.

## Ethics Statement

The studies involving human participants were reviewed and approved by the Joint Prevention and Control Mechanism of the State Council. Written informed consent to participate in this study was provided by the participants’ legal guardian/next of kin.

## Author Contributions

All authors read and approved the final version of the manuscript. QO, JML, and CL designed the study. CL, YZ, WY, QO, and JML have access to the raw data. TC, YZ, DY, JZ, CRL, YH, YY, and JWL contributed to the data analysis and interpretation. TC, YZ, QO, JML, and CL contributed to the drafting of the article. The corresponding authors attest that all listed authors meet authorship criteria and that no others meeting the criteria have been omitted. QO, JML, and CL are the guarantors.

## Conflict of Interest

The authors declare that the research was conducted in the absence of any commercial or financial relationships that could be construed as a potential conflict of interest.

## Publisher’s Note

All claims expressed in this article are solely those of the authors and do not necessarily represent those of their affiliated organizations, or those of the publisher, the editors and the reviewers. Any product that may be evaluated in this article, or claim that may be made by its manufacturer, is not guaranteed or endorsed by the publisher.
