# Supplementary material for: Nomogram Model for Prediction of SARS-CoV-2 Breakthrough Infection in Fujian: A Case–Control Real-World Study
Source: Front Cell Infect Microbiol. 2022 Jun 23;12:932204. doi: 10.3389/fcimb.2022.932204 (PMC9259977; doi:10.3389/fcimb.2022.932204)
Supplement: Supplementary file 1 [file DataSheet_1.docx]

**Supplement Table 1**. Difference analysis between the training cohort and the validation cohort

|  | Training cohort  n=4332 | Validation cohort  n=1857 | *P* values |
| --- | --- | --- | --- |
| SARS-CoV-2 breakthrough infection (%) |  |  | 0.740 |
| yes | 156 (3.6) | 63 (3.4) |  |
| no | 4176 (96.4) | 1794 (96.6) |  |
| Age (years old, %) |  |  | 0.619 |
| < 20 | 593 (13.7) | 261 (14.1) |  |
| 20-30 | 807 (18.6) | 335 (18.0) |  |
| 30-40 | 1265 (29.2) | 549 (29.6) |  |
| 40-50 | 897 (20.7) | 375 (20.2) |  |
| 50-60 | 570 (13.2) | 233 (12.5) |  |
| > 60 | 200 (4.6) | 104 (5.6) |  |
| Sex (%) |  |  | 0.449 |
| female | 2150 (50.9) | 1858 (52.0) |  |
| male | 2128 (49.1) | 892 (48.0) |  |
| Vaccine situation (%) |  |  | 0.998 |
| partially vaccinated | 515 (11.9) | 220 (11.8) |  |
| fully vaccinated | 3817 (88.1) | 1637 (88.2) |  |
| First dose brand (%) |  |  | 0.994 |
| Sinovac | 2249 (51.9) | 965 (52.0) |  |
| Sinopharm | 2083 (48.1) | 892 (48.0) |  |
| Second dose brand (%) |  |  | 0.580 |
| Sinopharm | 1803 (41.6) | 762 (41.0) |  |
| Sinovac | 2287 (52.8) | 979 (52.7) |  |
| unvaccinated | 242 (5.6) | 116 (6.2) |  |
| First dose time (days,%) |  |  | 0.420 |
| < 60 | 1363 (31.5) | 553 (29.8) |  |
| 60-120 | 1883 (43.5) | 825 (44.4) |  |
| > 120 | 1086 (25.1) | 479 (25.8) |  |
| Second dose time (days) |  |  | 0.850 |
| < 60 | 2237 (51.6) | 965 (52.0) |  |
| 60-120 | 1391 (32.1) | 601 (32.4) |  |
| > 120 | 704 (16.3) | 291 (15.7) |  |
| IC age (years old, %) |  |  | 0.593 |
| < 20 | 758 (17.5) | 335 (18.0) |  |
| 20-30 | 251 (5.8) | 102 (5.5) |  |
| 30-40 | 1255 (29.0) | 554 (29.8) |  |
| 40-50 | 1635 (37.7) | 702 (37.8) |  |
| 50-60 | 300 (6.9) | 106 (5.7) |  |
| > 60 | 133 (3.1) | 58 (3.1) |  |
| IC sex (%) |  |  | 0.833 |
| female | 2410 (55.6) | 1027 (55.3) |  |
| male | 1922 (44.4) | 830 (44.7) |  |
| IC vaccine situation (%) |  |  | 0.853 |
| partially vaccinated | 270 (6.2) | 111 (6.0) |  |
| fully vaccinated | 3140 (72.5) | 1341 (72.2) |  |
| unvaccinated | 922 (21.3) | 405 (21.8) |  |
| IC first dose brand (%) |  |  | 0.477 |
| Sinopharm | 1607 (37.1) | 664 (35.8) |  |
| Sinovac | 2097 (48.4) | 906 (48.8) |  |
| unvaccinated | 628 (14.5) | 287 (15.5) |  |
| IC second dose brand (%) |  |  | 0.621 |
| Sinopharm | 1436 (33.1) | 632 (34.0) |  |
| Sinovac | 1821 (42.0) | 756 (40.7) |  |
| unvaccinated | 1075 (24.8) | 469 (25.3) |  |
| IC first dose time (days,%) |  |  | 0.755 |
| < 60 | 1502 (34.7) | 659 (35.5) |  |
| 60-120 | 1536 (35.5) | 659 (35.5) |  |
| > 120 | 1294 (29.9) | 539 (29.0) |  |
| IC second dose time (days, %) |  |  | 0.775 |
| < 60 | 2082 (48.1) | 901 (48.5) |  |
| 60-120 | 1455 (33.6) | 607 (32.7) |  |
| > 120 | 795 (18.4) | 349 (18.8) |  |
| IC ORF1ab gene (Ct values) | 24.15 ± 6.37 | 24.13 ± 6.56 | 0.880 |
| IC N gene (Ct values) | 23.15 ± 6.48 | 23.11± 6.66 | 0.850 |

IC: index cases of vaccinated individuals.


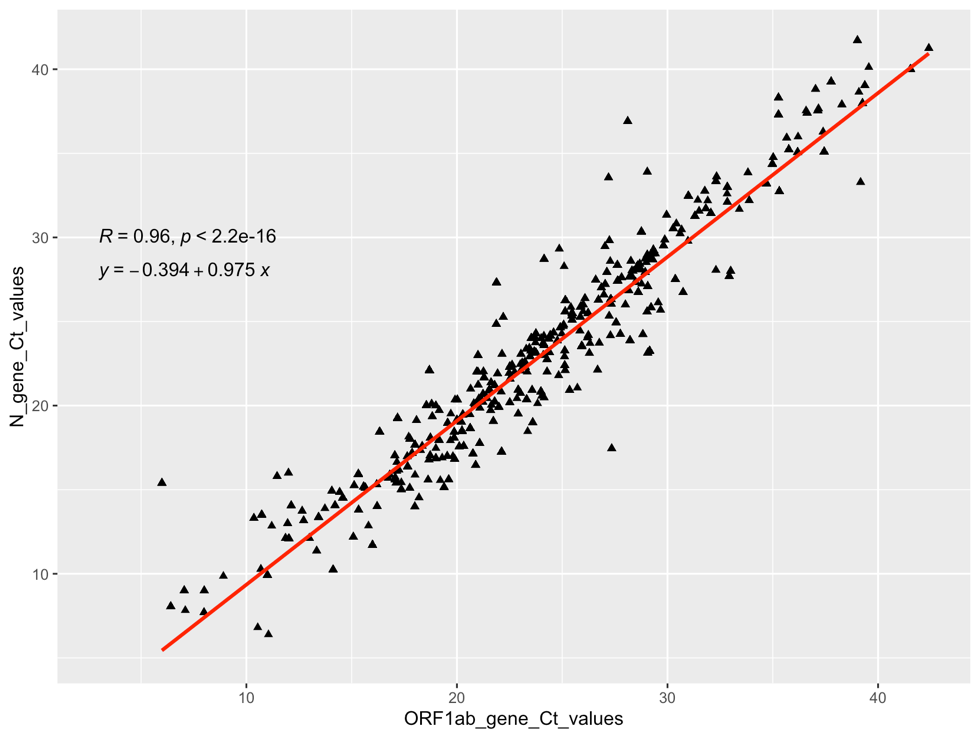


**Supplement Figure 1.** The correlation between ORF1ab gene Ct value and N gene Ct values was significantly high (*R*=0.958, *P*< 0.001) in index cases.
